# In Silico Gene Prioritization by Integrating Multiple Data Sources

**DOI:** 10.1371/journal.pone.0021137

**Published:** 2011-06-24

**Authors:** Yixuan Chen, Wenhui Wang, Yingyao Zhou, Robert Shields, Sumit K. Chanda, Robert C. Elston, Jing Li

**Affiliations:** 1 Department of Electrical Engineering and Computer Science, Case Western Reserve University, Cleveland, Ohio, United States of America; 2 Genomics Institute of the Novartis Research Foundation, San Diego, California, United States of America; 3 Infectious and Inflammatory Disease Center, Burnham Institute for Medical Research, La Jolla, California, United States of America; 4 Department of Epidemiology and Biostatistics, Case Western Reserve University, Cleveland, Ohio, United States of America; 5 Joint Institute of Systems Biology, College of Computer Science and Technology, Jilin University, Changchun, China; University of Swansea, United Kingdom

## Abstract

Identifying disease genes is crucial to the understanding of disease pathogenesis, and to the improvement of disease diagnosis and treatment. In recent years, many researchers have proposed approaches to prioritize candidate genes by considering the relationship of candidate genes and existing known disease genes, reflected in other data sources. In this paper, we propose an expandable framework for gene prioritization that can integrate multiple heterogeneous data sources by taking advantage of a unified graphic representation. Gene-gene relationships and gene-disease relationships are then defined based on the overall topology of each network using a diffusion kernel measure. These relationship measures are in turn normalized to derive an overall measure across all networks, which is utilized to rank all candidate genes. Based on the informativeness of available data sources with respect to each specific disease, we also propose an adaptive threshold score to select a small subset of candidate genes for further validation studies. We performed large scale cross-validation analysis on 110 disease families using three data sources. Results have shown that our approach consistently outperforms other two state of the art programs. A case study using Parkinson disease (PD) has identified four candidate genes (UBB, SEPT5, GPR37 and TH) that ranked higher than our adaptive threshold, all of which are involved in the PD pathway. In particular, a very recent study has observed a deletion of TH in a patient with PD, which supports the importance of the TH gene in PD pathogenesis. A web tool has been implemented to assist scientists in their genetic studies.

## Introduction

Dissecting genetic architectures of human diseases is a fundamental task in human genetics and has profound implications in biomedical research. However, great challenges exist because many common diseases are caused by multiple disease genes with small to moderate effects. Even diseases that show Mendelian inheritance may involve multiple genes due to heterogeneity. Gene-gene interactions, as well as gene-environment interactions, also play an important role in the development of diseases. Classifications of diseases, which are mostly based on observed phenotypes, may not necessarily reflect their underlying mechanisms. In addition, researchers have increasingly realized that there are many levels of controls along the paths from genotypes to phenotypes, resulting in a weaker relationship between genotypes and phenotypes [1] that may or may not be captured using traditional linkage or association approaches. Furthermore, linkage analysis usually can only identify chromosomal intervals that may contain up to hundreds of candidate genes owning to the limited number of crossovers in sampled families. Genome-wide association studies may also return many regions that show moderate to high signals. Experimental validations of so many candidate genes are usually beyond the ability of individual researchers owning to prohibitively high costs, both in terms of fund and time.

Another limitation of linkage or association studies is that their results only partially reflect the relationship between genes and traits on account of many reasons, such as small genetic effects, limited sample sizes, and limitations of statistical approaches. On the other hand, it is well understood that genes have to be transcribed and then translated into proteins, and proteins and other molecular entities have to function in a synchronized matter in the form of biological networks/pathways to perform normal functionalities or to cause pathological phenotypic changes. A variety of technologies exist to measure the levels of many such activities. Over the years, a vast amount of data from different sources has been accumulated and stored in a huge number of biological databases, many of which are publicly available. For a particular disease, such as breast cancer, tissue gene expression data might exist in some databases. Known disease genes and their interacting parters may have been recorded in protein-protein interaction (PPI) databases. Researchers may have also collected and constructed disease pathways based on previous studies. All these different data sets both confirm and complement each other, which helps researchers study the biological phenomenons from different aspects and levels. However, the conventional paradigm that aims to establish a direct relationship between genotypes and diseases through linkage and association studies mostly ignores all the intermediate processes and data associated with them.

To solve this dilemma, researchers recently have proposed approaches to prioritize candidate genes by using information from different data sources, such as sequence-based features [2], [3], functional annotation data [4], [5], protein interaction data [6]–[9], gene expression data [10], or a combination of multiple data sources [11]–[14]. The general idea of all these approaches is to rank candidate genes from linkage/association results according to their relationships with some known disease genes, reflected in these data sources. For many data sources, one has to measure the relationships between candidate genes and disease genes directly. For other data sources, such as PPI networks, one can either choose to measure the gene-gene relationships locally, or measure them globally. Köhler *et al*. [7] have shown that global measures perform better than local measures for prioritizing disease genes using PPI networks. A fundamental issue in studies using a single data source is the potential bias of their results caused by the incompleteness and noise of one particular data set. Intuitively, multiple data sources tend to provide better signal-to-noise ratio, and thus may improve prediction accuracy. ENDEAVOUR [11], [14] is a popular online gene prioritization tool that utilizes multiple data sources. It first ranks each candidate gene according to each individual data source using various metrics. The ranks from all data sources are then combined by using order statistics to obtain an overall rank. Though it might provide better results compared to approaches using a single data source, it has its own limitations. First, different metrics have to be derived for different data sources. It is not a trivial task if users need to add some new data sources that are not available from its web server. Second, for some data sources, such as PPI networks, simple local measures are used, which may provide inferior results as shown in [7]. In addition, each data source has its own noise or systematic errors. The ranks obtained by ENDEAVOUR from each individual data source are likely to be affected by those errors. When combining the ranks, such effects can hardly be evaluated or quantified.

**Figure 1:**
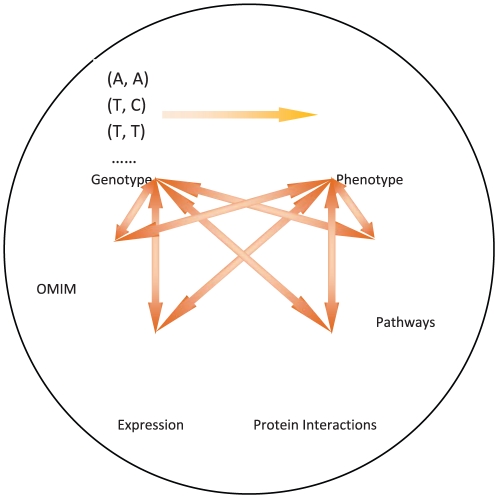
The proposed integrative framework.

In this paper, we propose a general framework (Figure 1) for candidate gene prioritization that can utilize multiple data sources by taking advantage of a unified graphic representation. Gene-gene relationships and gene-disease relationships are then defined for each network based on a global measure (*i.e.*, a diffusion kernel). These measures are in turn normalized to derive an overall measure across all networks, which is used to rank all candidate genes. For each candidate-disease gene pair, only the most informative network will contribute to the final gene-disease relationship. In this way, we can automatically minimize errors from unreliable data sources. We performed large scale cross-validation analysis on 110 disease families from the OMIM database using three data sources, based on protein interactions, gene expressions and pathway information. Results have shown that our approach consistently outperforms other two state-of-the-art programs (*i.e.*, random walk with restart [7] and ENDEAVOUR [11], [14]). We also confirmed that approaches based on global measures outperform approaches using local measures, and the performance of our approach improves with increase in the number of data sources. We have also defined a measure to quantify the informativeness of networks with respect to each disease. Improved performance has been observed on more informative diseases for all approaches. Based on the informativeness measure, we also propose an adaptive threshold score that can be used to select a small subset of candidate genes for further validation studies. Taking Parkinson disease (PD) as a case study, we tested our approach by considering all 3,243 genes that are shared by all three data sources. We identified four candidate genes (UBB, SEPT5, GPR37 and TH) that ranked higher than our adaptive threshold, all of which are involved in the PD pathway. In particular, a very recent study [15] has observed a deletion of TH in a patient with PD, which supports the importance of the TH gene in PD pathogenesis. A web tool has been implemented to assist scientists in their genetic studies, which can be accessed at http://cbc.case.edu/dir.

## Methods

### Data

#### Data Representation

One practical difficulty in integrating different data sources lies in the fact that different types of data are represented in different ways that are not directly comparable. To solve this problem, we consider each data source at a conceptual level. Essentially, we view a data source as evidence supporting relationships among genes. More specifically, for each gene pair, a data source can either support (to a certain degree) or not support the fact that these two genes have a relationship within the context of the given data. This is apparent in terms of PPI networks. A direct interaction between a pair of proteins either has been observed or has not been observed yet. The relationships between a candidate gene (encoding the corresponding protein) and all other genes/proteins can be thus defined. Such information can also be obtained from other data sources. For example, gene expression data can be transformed into gene co-expression networks by connecting genes with similar expression patterns. To represent known knowledge from biological pathways, a simple network can be built by connecting genes (or their products) that coexist in any pathway. Co-existence networks can also be built from other data sources, such as text. In such a representation, each data source is encoded by a graph, where nodes represent genes and edges (with possible weights) represent relationships between genes. It is obvious that such a representation only partially captures information from original data sources and inevitably inherits incompleteness and noise from its original data. However, information loss as well as noise can be assumed to be independent for the different data sources. Our hypothesis is that, when one observes strong evidences from multiple sources using this graph representation, it implies a possible true signal that is worth further investigation. In this work, we primarily focus on three specific data sources, namely, PPI, gene co-expression and pathway networks. Knowledge from mining the literature is not considered directly because it is known that methods relying on text mining may produce biased results [7].

#### Protein-Protein Interaction Data

The protein-protein binding data used in this study were derived from the HyNet yeast-two-hybrid database [16] and curated molecular interaction databases including Reactome [17], BIND [18], MINT [19] and HPRD [20]. Duplicated edges between the same pair of nodes were combined and edges connecting a node to itself were deleted. The final protein-protein interaction network contains 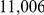 human genes that encode proteins in the network and 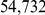 edges. This exact dataset has been used in other previous biological studies [21], [22].

#### Human Gene Expression Data

The human tissue expression dataset was obtained from GNF's SymAtlas web site [23]. This dataset consists of 79 human tissues in duplicates, measured using the Affymetrix U133A array that consists of 22,215 probe sets. All array measurements were processed and normalized using the Affymetrix MAS5 algorithm. Pairwise Pearson correlation coefficients were calculated and a pair of genes were linked by an edge if their correlation coefficient is greater than 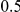. The correlation coefficients were then assigned as weights for edges. The final network consists of 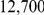 genes and 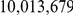 edges among them.

#### Pathway Data

The pathway dataset was obtained from the Kyoto Encyclopedia of Genes and Genomes (KEGG) [24] pathway database, which is a collection of manually curated biological pathways. For simplicity, an edge was constructed between two genes (or gene products), if they coexist in any pathway. The “pathway network” constructed this way consists of 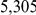 nodes and 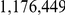 edges.

#### Known Disease-Gene Associations

OMIM [25] is a large database about genes and disease phenotypes curated by domain experts. We have extracted the disease-gene relationships using the software BioMart [26]. In addition, Köhler *et al*. [7] have investigated similarities among diseases based on the entries in OMIM and classified those with similar or even indistinguishable phenotypes into disease families. By doing so, the number of disease genes per family will be much greater than the number of genes per disease. We adopted this classification of diseases and further updated the disease families with new information by adding newly discovered disease genes since Köhler *et al*. 's paper was published. There are total 944 distinct genes from 110 disease families. The largest family contains 44 genes whereas the smallest one contains 3 genes. The average number of genes per family is 8.58.

### Approach

#### Candidate Gene Ranking Using a Single Source

Once the information from a data source is represented by a network, the relationship between a candidate gene and a disease can be measured by the relationship between the candidate gene and all known disease genes. The basic assumption of the Guilt-by-Association principle [27] is that genes that are “close” to each other in a network are expected to perform similar functions, thus genes that are closer to disease genes will be more likely to be associated with the same disease, and they should be ranked higher. This principle is largely true for many networks, such as PPI networks, and has been validated by many previous studies. To define the closeness of a pair of genes or one gene to a group of genes in general, several distance/similarity measures have been proposed by considering the topology (as well as edge weights when possible) of a network, either locally (such as direct neighbor(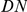), shortest path(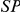)) or globally (such as diffusion kernel (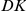) and random walk with restart (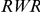)). All of these measures have been used in previous studies (*e.g.*, in [7]). For the sake of completeness, we briefly introduce them here and show how they can be used in gene ranking. We will compare the performance of our proposed approach with these methods.

Let 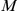 denote the adjacency matrix of a given network. For an unweighted network such as the PPI or the pathway network, 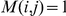 if there is an edge between gene 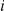 and gene 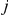, and 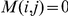 otherwise. For a weighted network such as the co-expression network, 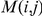 is the Pearson correlation coefficient of the two genes 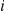 and 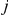 if their correlation is greater than 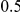, and 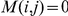 otherwise. Let 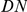, 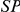 and 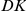 denote the pairwise distance/similarity matrix for measures based on direct neighbor, shortest path and diffusion kernel, respectively. The direct neighbor distance 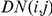 between two genes 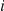 and 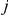 is defined as 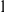, if 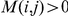, and 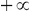 otherwise. The shortest path distance 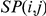 between two genes 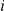 and 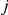 is defined as the length of a shortest path between the two genes, which can be easily calculated based on standard graph algorithms. The diffusion kernel is defined as: 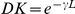, where 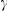 is a tuning parameter and 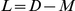, 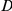 being a diagonal matrix with the diagonal elements containing the node degrees. The diffusion kernel represents a global similarity between nodes in a graph, with higher values representing closer relationships. For nodes that are not connected, their values will be 0. For a specific disease family 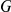 with a set 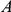 of known disease genes, and for a candidate gene 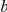 in a set 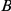 of candidate genes, the relationship between 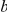 and 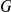 is represented by the average distance between 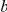 and all known disease genes in 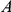. For example, for the 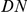 measure, 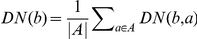. Such a proximity score can then be used to rank all the genes in 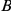.

Different from the three measures defined above, the 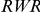 approach [7] directly defines the relationship of a gene with a group of disease genes. It is described as an iterative walker's transition from its current node to a randomly selected neighbor starting at a set of given seed nodes (disease genes). Formally, the 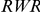 is defined as: 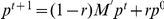, where 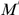 is the column-normalized adjacency matrix 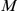 and 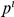 is a vector where the 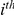 element holds the probability of being at node 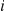 at time step 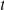. The initial probability vector 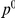 is constructed such that equal probabilities are assigned to the nodes in set 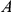, with the sum of the probabilities equal to 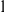. The parameter 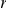 represents the restart probability. The proximity score of a candidate gene 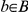 is then defined as the corresponding element in the steady-state probability vector 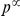, which is usually approximated by 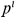 when 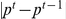 is smaller than a predefined threshold. K
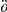
hler *et al.* [7] compared the performance of these four measures in prioritizing candidate genes using the PPI network. They showed that the two global measures (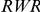 and 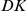), which incorporate all the connectivity information in a network and have similar performance, clearly outperformed the two local measures (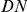 and 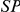).

#### Integrating Multiple Sources

Significant challenges exist in integrating different data sources, even if they all have been represented using networks, because the distances defined in different networks may not be directly comparable. In this study, we propose an importance measure that is defined based on the relative strength of the distance between a pair of genes among all pairwise distances within each network. On assuming different networks are independent, these measures from different networks can be directly compared with one another. Such a framework can be applied to any measures that can define pairwise distances/similarities, such as direct neighbor, shortest path and diffusion kernel. However, it cannot be directly applied to the 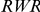 [7]. Because global distance measures are much better in capturing the overall relationships in a network, we mainly focus on the framework in combination with the diffusion kernel approach. More specifically, let 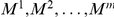 denote the adjacency matrices derived from 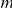 different datasets, respectively. Let 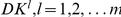 denote their diffusion kernels. The importance of the similarity between a gene pair 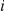 and 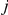 is defined as:
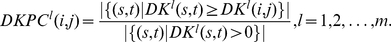
The numerator measures the number of pairs that are closer than the pair 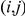. The denominator counts the total number of connected pairs. Intuitively, for each gene pair, its 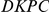 value is equal to one minus the percentile of its original diffusion kernel similarity among all connected pairs. Therefore, the value is smaller (or more significant) when the two genes are more similar. If gene 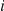 and gene 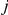 are not connected in network 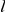, 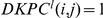. With this definition, all relationships between pairs of genes are scaled between 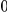 and 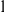 for all networks and can be compared across different networks. Based on this importance score, we further define our final data integration rank (DIR) score for each candidate gene 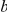 from 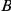 with respect to a specific disease family 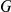 with a set 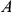 of known disease genes as:
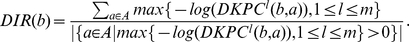
The numerator sums the evidence over all disease genes within the disease family. And for each disease gene 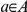, it chooses the most informative network to use by taking the *max*. The denominator just counts the number of disease genes that provide information in the numerator (*i.e.*, those that are connected to the candidate gene). This score reflects the overall relationship between gene 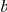 and all known disease genes in 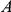. By taking the *max* instead of average, it potentially yields better performance because when some networks are incomplete, which happens frequently, the average score is usually much lower. The 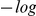 is mainly for the stability of the score. The normalization by dividing the number of disease genes that provide information can further account for the incompleteness of some networks.

#### Meta Score and Declaration of Positives

One can directly use the 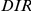 score defined above to select genes that might be associated with diseases. The greater 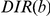 is, the more likely gene 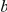 will be associated with the disease and it will have higher rank. Conventionally, researchers select a fixed number of candidate genes (so called top-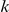 approach) to report prioritization results for all disease families. However, different disease families usually have different numbers of known disease genes. It may not be appropriate to use a global threshold in such a case. Following the idea proposed by Zhou *et al.* [28], we define and automatically calculate a meta score 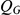 for a specific disease family 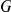 with a set 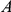 of known disease genes based on the relationships of all these known disease genes in all networks. Let 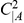 denote the binomial coefficient with parameters 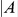 and 2. 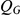 is defined as:
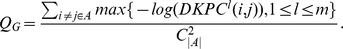
Intuitively, the meta score 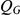 measures the average “closeness” or significance of all disease genes of this disease family from all the networks. If a candidate gene is closer to the disease genes than the disease genes are to themselves on average, this candidate gene is more likely to be associated with the disease, too. This meta score can be used as a threshold for declaring significant candidate genes. In the Results section, we will discuss the use of 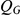 and its variants as “adaptive ranking thresholds” and evaluate their performance in comparison with the top-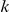 approach.

### Informativeness of a Network

The informativeness of networks is different for different disease families. Even though the networks are quite comprehensive, some disease genes may not occur in a network at all, or may have limited connections. Therefore, for some disease families, it is not appropriate to use the data sources to prioritize genes if the networks themselves do not contain enough information about these disease families. To formally quantify the informativeness of a network with respect to a disease family 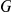, we define a measure of informativeness 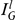 of a network 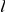 for a disease family 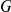 with a set 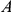 of disease genes as the average pairwise relationship between known disease genes:
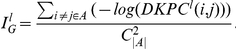
In our experiments below, in addition to the overall performance using all disease families, we also perform evaluations by separating the disease families according to their informativeness.

### Validation Method and Evaluation Criteria

We evaluate the proposed method using the leave-one-out cross-validation approach, which has been adopted by many previous studies (*e.g.*, [7]). Briefly, for each disease gene in each of the 110 disease families, we obtain 100 genes located nearest to this disease gene on the same chromosome and rank all of them together with this disease gene according to the score defined above. The process is repeated for all disease genes to obtain final results. We use two measures to measure the performance of our approach. First, for each run, the enrichment factor is defined as 50/(rank of the tested disease gene), which will be highest if the tested gene ranks first. Second, we also use the measure of the receiver operating characteristic (ROC) curve, which shows the relation between the sensitivity (true positive) and the specificity (true negative rate) by varying the threshold for declaring positives. The area under the ROC curve (AUC), which provides an overall measure of the performance, is used to compare different approaches.

## Results

We first constructed the gene co-expression network, the PPI network and the pathway network as described earlier, and calculated the 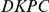 scores for each of them as the knowledge base of our approach. We performed extensive experiments to test the performance of our proposed approach under different scenarios and compared its performance with two existing cutting-edge approaches, RWR [7] and ENDEAVOUR [11], [14]. We first evaluated the performance of our measure and all the three other measures (*i.e.*, 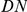, 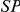, and 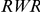) on a single network, followed by the experiments using different number of networks. We then compared our results with those by ENDEAVOUR using three similar networks. We also examined the results by separating the disease families according to their mechanisms and their informativeness. Lastly, as a test case, we present our results on the Parkinson disease family. The approach has been implemented as a web tool and can be accessed freely.

### Performance Using All Disease Families

**Figure 2:**
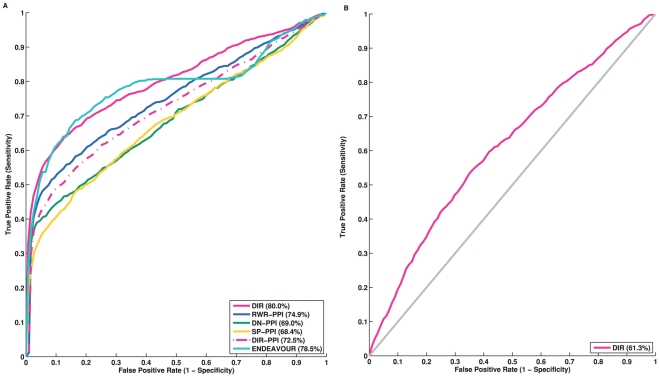
A: ROC curves of cross-validation results by different approaches. The suffix “-PPI” after each method indicates it uses the PPI network only. B: The ROC curve of DIR using the re-wired networks.

By using the leave-one-out cross-validation, we first compared the performance of our algorithm on all of the updated 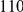 disease families with several state of the art algorithms that utilize single as well as multiple data sources. More specifically, we tested our approach (DIR) on the three networks that we constructed. Results from ENDEAVOUR were obtained from three comparable data sources that were listed in their package (*i.e.*, PPI from HPRD, pathway from KEGG and the same expression data from Su *et al.* [23]). We also included the three approaches (RWR, DN and SP) as well as our own approach on the PPI network alone (denoted as DIR-PPI). The PPI network was chosen in the study of performance on a single network because it has higher coverage and is more informative than the other two networks, and PPI networks have been widely used in previous studies (*e.g.*, [21], [22]). In our implementation, if the disease gene left out for testing is not in any network, it was assigned a random rank between 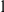 and 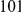. Figure 2A shows the ROC curves of all the approaches tested. The AUC values are also listed (in parenthesis) for each method. It is apparent that DIR has the best overall performance, with the AUC around 80.0%. The two approaches DIR and ENDEAVOUR, using multiple data sources outperform all the approaches using the PPI network alone. This is consistent with the general belief that by collecting more evidences from different data sources, the prediction results can be improved. The significant improvements of DIR compared to DIR-PPI, as well as to RWR, further illustrate the value of integrating multiple data sources. Though DIR is only slightly better than ENDEAVOUR in terms of the AUC values (80.0% *vs.* 78.5%), the total number of tested genes that were ranked first by DIR is much greater than the number of first ranked genes by ENDEAVOUR (330 *vs.* 243). Consequently, the enrichment factor achieved by DIR is better than that of ENDEAVOUR (21.9 *vs.* 18.5). The flat area in the middle of the ROC curve generated by ENDEAVOUR is due to the way it deals with missing information (see supplemental materials of [11]). On a single network, the two approaches incorporating the global topology (RWR and DIR-PPI) outperform the two approaches using local measures (DN and SP). RWR is slightly better than DIR-PPI, which is also consistent with previous studies [7]. Therefore, we dropped the three approaches using a single network (DIR-PPI, DN and SP) from further comparisons.

In general, disease genes usually receive more attention and usually have been studied more intensively after they were discovered. This is reflected by the fact that normally the average degree (*i.e.*, the number of links) of disease genes in some networks is much greater than the average degree of non-disease genes (*e.g.*, 15.5 vs 9.5 in the PPI network). To assess whether our method critically relies on this degree bias, we randomly shuffled the networks while keeping the degree of each node unchanged. We performed the same leave-one-out experiment. Roughly speaking, the results (Figure 2B) show that the ROC curve is close to the diagonal of the coordinate plane, which illustrates that our results were not driven by the underlying degree distribution. However, the AUC based on re-wired networks is not 0.5, which suggests some bias that may be due to other reasons. We suspect the density of the expression and pathway networks might affect this result.

#### Parameter Tuning

**Figure 3:**
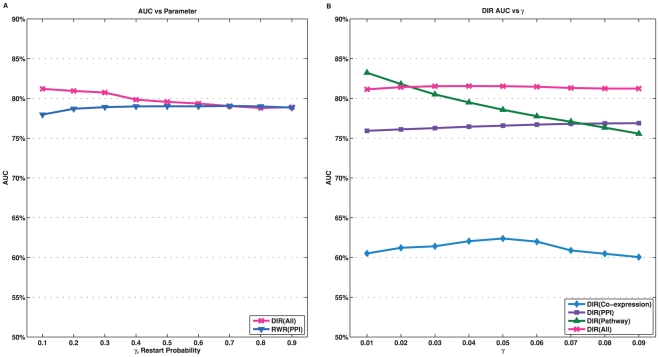
Robustness assessments of DIR and RWR for their parameters ranging from 0.1 to 0.9 (left), as well as DIR from 0.01 to 0.09 (right).

Notice that both DIR and RWR have some user-defined parameters in their framework. We performed robustness analysis of both approaches and the results presented above were obtained using the parameters that achieved the best performance for both approaches. More specifically, the RWR method has a parameter 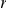 that indicates the restart probability. We varied 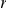 from 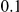 to 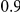 at increments of 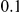. The best result was obtained when 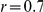. Therefore, we fixed 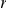 at 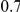 in our experiments. DIR has a parameter 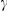. We tested 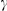 in the same manner from 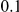 to 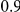. The best result was obtained when 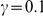 (Figure 3A). We further tested the performance of DIR for 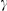 from 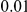 to 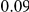 at increments of 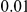 for all three networks together and separately. No significant changes were observed (Figure 3B). Overall, the performance was very robust to 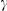. We selected 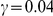 in our experiments. When we performed the analysis on each network individually here, only genes that were in the network were considered. This was different from the experiment using all networks, as well as the experiments using single networks elsewhere, in which cases all genes in a defined control set were considered and a random rank was assigned to a gene not in a network. When ignoring missed genes, using the pathway alone actually can achieve better results when 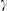 is small (Figure 3B), which is consistent with the fact that tight/direct links in the pathway network are much more important than indirect links.

#### Performance Using Alternative Control Sets

**Figure 4:**
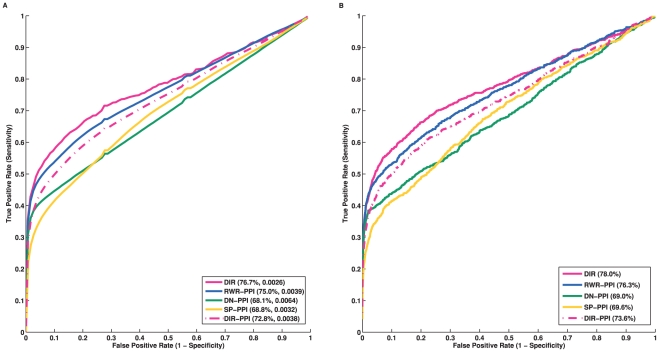
Left: The average performance of the five approaches using 100 randomly selected control sets. Right: The performance of the approaches using all genes in the PPI network as the control set.

In our experiments, we selected the 100 closest genes for each disease gene as its control set. In order to test the robustness of our approach with respect to the selection of control sets, we performed large scale cross-validation experiments using two alternatives. In the first experiment, for each disease gene, we randomly selected 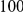 genes from the PPI network as the control set. We performed the leave-one-out cross-validation and obtained the performance result of each approach. We further repeated this procedure 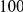 times to obtain the variance of the AUC values. Results show that the variances of the AUC values of all approaches tested are very small and our method consistently performs better than RWR and other approaches based on local measures (Figure 4A). The average performance of DIR using control sets from the PPI network is not as good as its performance using the closest neighboring genes. We suspect this is mainly caused by the missing of some neighboring genes in these networks. In the second experiment, we examined the performance of these approaches using a genome-wide control set. We took all the genes in the PPI network excluding those disease genes as the control set. Once again, the leave-one-out cross-validation was performed. Our method again consistently performs better than RWR and other approaches based on local measures (Figure 4B). Owing to its efficiency issue, ENDEAVOUR could not finish the analysis on these two experiments in several days, therefore we could not obtain its results.

### Performance on Different Categories of Diseases

**Figure 5:**
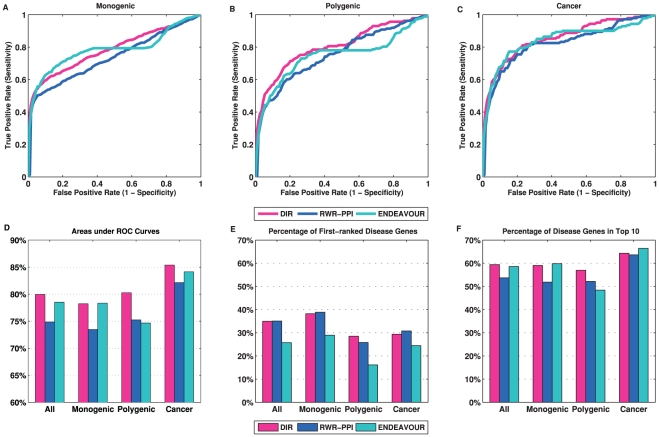
Cross-validation results of three approaches on different disease categories. (A) ROC curves for monogenic diseases. (B) ROC curves for polygenic diseases. (C) ROC curves for cancers. (D) AUC values on all disease families and on the three categories. (E) Percentage of first-ranked disease genes for all diseases and the three categories. (F) Percentage of disease genes ranked in top-10 for all diseases and the three categories.

On the basis of the mechanisms of diseases, Köhler *et al*. [7] separated the 110 families into three categories: namely, monogenic diseases, polygenic diseases, and cancers. The number of families and the number of total disease genes in each of the three categories are 85/615, 13/186, 12/143, for monogenic, polygenic, and cancer diseases, respectively. We evaluated and compared the three approaches (DIR, RWR and ENDEAVOUR) over the three categories of disease families separately. DIR achieved the best overall performance and outperformed both RWR and ENDEAVOUR in all three categories (Figure 5A–D) in terms of the AUC values. Interestingly, all three approaches have the best performance (*i.e.*, best AUC values) for the cancer disease families (Figure 5D). DIR performed much better than RWR and ENDEAVOUR for the polygenic disease families, while DIR and ENDEAVOUR performed much better than RWR for the monogenic diseases. In terms of the fraction of disease genes ranked in the first place (Figure 5E), both DIR and RWR had about 35% of all tested genes ranked first, while the fraction of first ranked genes by ENDEAVOUR was much lower (about 25%). Similarly, when separated into three categories, the fraction of genes ranked first by ENDEAVOUR was much smaller than those of DIR and RWR. ENDEAVOUR was able to catch up in terms the number of genes ranked in the top ten list (Figure 5F), which explains why it has better overall AUC than RWR. For different disease categories, all approaches had better results for the monogenic diseases when considering the first ranked genes. The highest enrichment factor was achieved by DIR in the monogenic disease families (23.0) and the lowest was ENDEAVOUR in the polygenic diseases (13.8).

### Informativeness of Networks and Performance Using Different Numbers of Networks

We advocate the use of our approach for its capability of being able to incorporate multiple data sources when prioritizing candidate genes. To explore this further, we evaluated the informativeness of the three networks with respect to the disease families using the measure defined earlier, and examined the performance of our approach using different combinations of data sources. First of all, DIR has shown consistent improvements for all the measures (the AUC values, the number of first-ranked disease genes, the number of disease genes in the top-10 highest ranked genes, and the average enrichment factors) when increasing the number of data sources (Table 1), which again verified our hypothesis that the approaches with multiple data sources are preferred in gene prioritization. Second, among the three networks, the gene co-expression network was the least informative one, which is consistent with observations from previous studies (*e.g.*, [29]) that physical interaction data including PPI usually provides stronger evidence for gene function predictions compared to expression correlation. It seems counter intuitive that the PPI was more informative than the pathway network. This is mainly due to the difference in size/coverage of the two networks. The number of genes in the pathway network is significantly less than the number of genes in the PPI network. Disease genes not in the pathway network received a random rank, which contributed to the relative low performance of the pathway network. When only considering genes that appear in the pathway network, the pathway network is actually more informative (*e.g.*, see Figure 3B). The combination of the PPI network and the pathway network performs very well. Overall, the three networks together show the best performance. Although the gene co-expression network is not very informative as the PPI network and the pathway network, including it increases the coverage of genes and thus enables prioritizing candidate genes not captured by the other two networks.

**Table 1:** Cross-validation results using different combinations of data sources.

Data Source	EXP	PWY	PPI	EXP+PWY	EXP+PPI	PPI+PWY	ALL
AUC	58.3%	64.8%	72.5%	71.9%	76.5%	77.3%	80.0%
Ranked first	57	175	278	179	291	320	330
In top-10	199	339	466	391	513	520	561
Enrichment	6.7	13.5	18.7	13.7	19.9	21.1	21.9

EXP: co-expression network, PPI: protein-protein interaction network, PWY: pathway network.

The results above have shown the overall improvement of DIR when including more data sources. To further showcase the improvement of prioritization on specific disease families by integrating more data sources, we calculated the informativeness of each network with respect to each disease family (*i.e.*, 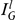). We selected three disease families as an example to show the improvement by approaches using multiple data sources (Table 2). The informativeness of networks on all diseases can be found in Dataset S1. In one example, the disease family “Generalized epilepsy with febrile seizures plus” obtains little information from the PPI network. Therefore it was not surprising that the RWR, which depends on the PPI network solely, could not correctly predict disease genes in the cross-validation. In contrast, the gene co-expression network provided sufficient information about their connections. Consequently, the two approaches DIR and ENDEAVOUR using the gene co-expression network returned much better results. In another example, the disease family “Pituitary dwarfism” has strong information from the PPI network and has little information from the other two networks. All three approaches performed well on this family, which also illustrated that the performance of both DIR and ENDEAVOUR were not weakened by including more networks, even if some of them were not informative. In a last example (Aicardi-Goutieres syndrome), both the gene co-expression network and the pathway network contributed to the success of DIR in ranking the three genes. Relying on the PPI network alone, RWR could not successfully rank these genes and missed one gene (RNASEH2C) because it was not in the PPI network.

**Table 2:** Three examples show improvements of DIR by integrating multiple data sources.

Disease Family/Informativeness			Gene Name (Entrez ID)	DIR	RWR	ENDEAVOUR
Generalized epilepsy with			SCN2A(6326)	6	66	1
febrile seizures plus			SCN1A(6323)	7	-	2
Exp	PPI	Pathway	SCN1B(6324)	7	61	1
4.09	0.10	0.46	GABRG2(2566)	6	62	4
			GABRD(2563)	1	50	7
Pituitary dwarfism			LHX3(8022)	1	1	4
Exp	PPI	Pathway	POU1F1(5449)	1	1	3
0.90	8.08	0.00	HESX1(8820)	1	1	1
			PROP1(5626)	1	1	1
Aicardi-Goutieres syndrome			RNASEH2A(10535)	1	29	23
Exp	PPI	Pathway	RNASEH2B(79621)	2	72	16
2.29	0.31	4.10	RNASEH2C(84153)	1	-	72

The first column also lists the informativeness of each network contributing to each disease family.

### Performance on Informative Diseases

**Figure 6:**
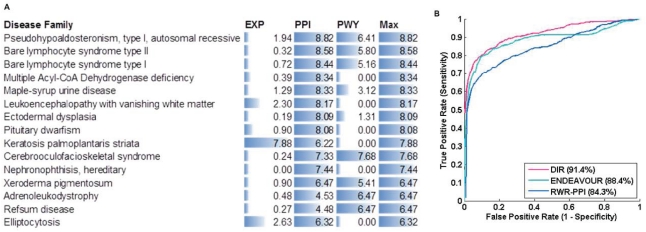
A: A partial list of disease families that are most informative. B: Cross-validation results excluding disease families with 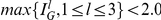.

When using other data sources to prioritize candidate genes for a disease, the effectiveness of any approach is essentially determined by the coverage and information content in those data sources, which represents the existing knowledge about the disease. Based on the network informativeness (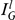), we ranked the disease families according to the maximum value of the informativeness of the three networks. We chose a subset of diseases that were more informative, defined as 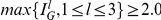 (which roughly corresponded to an average 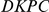 score of 0.01 or lower). There was a total of 66 such families, consisting of 490 disease genes, and the top 15 families are listed in Figure 6A. The list of all disease families can be found in Dataset S1. We summarize the cross-validation experiment results of the three approaches again but using only this set of 66 families (Figure 6B). Apparently, the performance of all three approaches improved dramatically. For example, the AUC values increased significantly: from 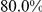 to 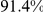 for DIR, 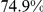 to 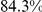 for RWR, and 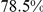 to 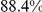 for ENDEAVOUR. This suggests that with more information available, network-based approaches can make better prioritization. Researchers can always first evaluate the informativeness of the networks with respect to their own diseases before applying any *in silico* gene prioritization approaches.

### Performance Using an Adaptive Rank Threshold

After obtaining a ranked list of all candidate genes, one needs to define a rank threshold to declare disease susceptibility genes for further studies. Ideally, such a threshold should be able to capture the true disease genes while keeping the number of non-disease related genes as small as possible. In practice, one has to balance between the True Positive Rate (TPR) and the False Positive Rate (FPR). To increase the TPR, one may always increase the FPR. A straightforward method to declare positives is the Top-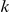 criterion (*e.g.*, 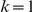 or 10) that declares all the top 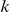 best ranked candidate genes as disease susceptibility genes. Our framework can naturally utilize the meta score 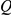 (*i.e.*, 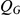 for disease 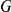) as the selection criterion. The 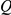 score reflects the relationship between known disease genes. Our hypothesis is that the relationship between a disease susceptibility gene and known disease genes should be similar to the relationship among known disease genes themselves. Our approach ranks candidate genes together with known disease genes as well as with the meta score 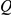. If a candidate gene is ranked better than 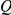, it is likely to be a true disease gene given that 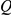 is also ranked relatively high. In the case that no candidate gene is ranked better than 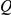, we declare the first ranked candidate gene as the disease susceptibility gene. We call such a criterion the “Q+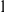
” rule. In some cases, the relationship among existing disease genes is not so strong, resulting in a low 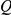 score. To avoid too many false positives, we use the 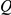 score only if it itself ranks in the top-10 (excluding known disease genes). We call this one the “Q+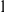
OR
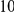
” criterion. We have evaluated the Top-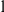, Top-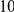, Q+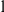, and Q+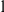
OR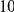 criteria on the 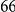 informative disease families defined above. We calculated the TPR as the ratio of successfully detected disease genes out of the total number of disease genes. The non-disease genes that ranked higher than each criterion are the false positives. The FPR is calculated as the number of false positives divided by the total number of candidate genes. Table 3 shows the TPR and FPR under each of the four criteria. Although the Top-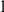 criterion has the smallest FPR, it also suffers from the smallest TPR. On the contrary, the Top-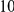 criterion gives the highest TPR, but also the highest FPR. Our criteria Q+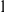 and Q+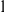
OR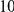 lie in between the two. In particular, the performance of Q+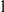
OR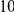 is appealing. Compared to the Top-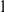 criterion, it can actually increase the TPR by 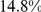 while only increasing the FPR by 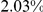.

**Table 3:** True Positive Rate and False Positive Rate using different criteria.

Criterion	Top-1	Q+1	Q+1OR10	Top-10
True Positive Rate	54.0%	68.8%	68.8%	81.6%
False Positive Rate	0.46%	3.64%	2.49%	9.18%

### A Case Study

**Figure 7:**
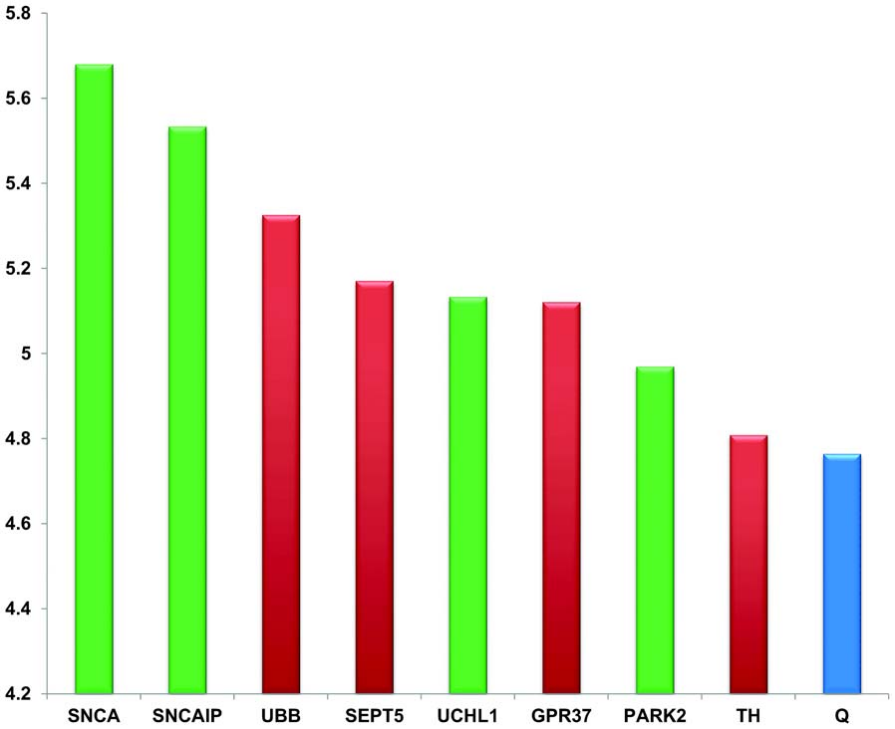
In the case study of the PD disease family, four candidate genes (in read) and four disease genes (in green) ranked higher than the 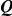 score (in blue), all of which are ordered according to their 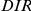 values.

**Figure 8:**
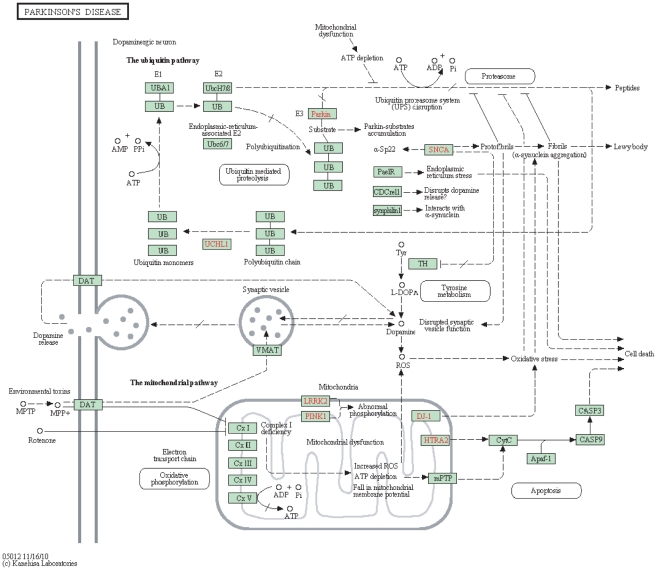
The PD pathway obtained from the KEGG pathway database.

We chose the disease family “Parkinson Disease” (PD) as a case study to perform a large scale *de novo* test of our proposed algorithm. Parkinson disease is one of the most common neurodegenerative disorders. For the PD disease family, we have used the same definition in Köhler *et al*. [7], which consists of several forms of Parkinson diseases such as, PARK, PARK1, PARK2 (See Table 4 for details). The disease family has 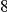 known disease genes and the cross-validation experiment ranked seven of them at the first place and one of them (LRRK2) at the second place. To identify some potential new PD disease genes, we constructed the candidate gene set by including all 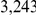 genes that have appeared in all three networks. We ranked the candidate genes together with the known disease genes and used the 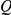-score to declare positives. Taking all 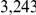 genes together, the 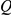-score ranked number 9, and 4 disease genes and 4 candidate genes had higher scores than 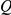 (Figure 7). The four candidate genes are ubiquitin B (UBB), septin 5 (SEPT5), G protein-coupled receptor 37 (GPR37) and Tyrosine hydroxylase (TH), all of which have been involved in the Parkinson disease pathway (Figure 8). UBB encodes ubiquitin, one of the most conserved proteins known. Ubiquitin is required for ATP-dependent, nonlysosomal intracellular protein degradation of abnormal proteins. Aberrant forms of this protein have been noticed in patients with Alzheimer and Huntington diseases [30], but not PD, though all three diseases share a common feature in the accumulation of insoluble protein deposits. SEPT5 is a member of the septin gene family of nucleotide binding proteins, which is shown as CDCrel1 in the PD pathway (Figure 8). GPR37 is a substrate of parkin (PARK2), and its insoluble aggregates accumulate in brain tissue samples of Parkinson's disease patients [31] (shown as PaelR in Figure 8). The protein encoded by TH is involved in the conversion of tyrosine to dopamine. It is the rate-limiting enzyme in the synthesis of catecholamines, hence plays a key role in the physiology of adrenergic neurons. Mutations in this gene have been associated with autosomal recessive Segawa syndrome. Missense mutation in both alleles of the TH gene is known to cause dopamine-related phenotypes, including dystonia and infantile Parkinsonism. Most recently, a study has found a rare novel deletion of the entire TH gene in an adult with PD [15]. The result from this study had not been entered into the OMIM database. This clearly shows the value of our *in silico* prioritization approach, and the top ranked genes returned by our approach should receive more attentions in follow-up or validation studies. We have also tested RWR and ENDEAVOUR on the same data set. All the four genes reported by DIR are in the top 10 list of ENDEAVOUR, and five other genes in the top 10 list of ENDEAVOUR are also ranked high by DIR (*i.e.*, in top 25 among more than 3000 candidates). The other gene, ALS2, ranked number 2 by ENDEAVOUR, is not in the top 100 by DIR. Literature search reveals that ALS2-related disorders include Autosomal Recessive Juvenile Amyotrophic Lateral Sclerosis, Infantile-Onset Ascending Hereditary Spastic Paralysis and Juvenile Primary Lateral Sclerosis, but not PK. Results from RWR are quite different from DIR and ENDEAVOUR, which is not surprising given that RWR has only utilized the PPI network. The top 100 genes from each method can be found in the supplemental Dataset S2.

**Table 4:** Disease genes from the Parkinson disease family and related disorders.

Genes (OMIM ID)	Disorder (OMIM ID)
SNCA (168601)	Parkinson disease , familial, type 1 (PARK1) (163890)
PARK2 (600116)	Parkinson disease 2, AR, juvenile (PARK2) (602544)
UCHL1 (191342)	Parkinson disease 5 (191342)
PINK1 (605909)	Parkinson disease 6, AR, early-onset (608309)
PARK7 (602533)	Parkinson disease, autosomal recessive, early-onset (606324)
LRRK2 (607060)	Parkinson disease 8 (609007)
HTRA2 (610297)	Parkinson disease 13 (606441)
SNCAIP (603779)	Parkinson disease (603779)

## Discussion

In this paper, we have proposed a candidate gene prioritization approach that can integrate multiple data sources by taking advantage of a unified graphic representation of information. Our results have shown that based on a single network, both our approach and the RWR approach have better performance than measures based on local topology (*i.e.*, 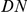 and 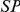), which is consistent with observations made by previous studies. Our experiments have also shown that by integrating multiple sources, DIR significantly outperformed all approaches relying on single sources. Consistent improvements have been observed for DIR when increasing the number of data sources from one to three. Using three data sources and large scale cross-validations, we have shown that the proposed approach outperforms two cutting-edge methods. In terms of the AUC values, the improvement of DIR over RWR is more impressive than the improvement of DIR over ENDEAVOUR. Actually, in both cases, the improvements should be statistically significant. Though one cannot directly estimate the errors for these experiments, robustness analysis using different control sets have shown that the estimated standard error of DIR is very small (0.0026, Figure 4A), almost an order of magnitude smaller than the performance difference. Furthermore, the fraction of first ranked genes by DIR is much greater than the fraction by ENDEAVOUR. The improvement of DIR over RWR can be attributable to the inclusion of more data sources. Comparing to ENDEAVOUR, in which case it first ranks a gene based on an individual data source, the definition of the 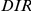 score, which utilizes only the most informative network for each individual disease gene, may give us some advantage.

We have also presented an adaptive threshold to automatically select a small subset of most promising candidate genes, which can significantly improve the true positive rate while keeping the false positive rate low. Our results have confirmed that global measures are better than local measures in capturing gene-gene relationships. Based on a global measure of gene-gene relationship, we have proposed a measure of network informativeness, which can be used to guide gene prioritization studies. We have shown that the accuracy of our approach has been improved when using data with higher quality. A case study on Parkinson disease has illustrated the potential of the proposed approach.

The framework can be easily extended to include more data sources, as long as there is an appropriate definition of gene relationships for each data source. On the other hand, it is not always easy to capture all the information from some original data sources by using a graph representation. We will investigate the inclusion of more data sources in our future work. For a specific disease, the prediction result will be limited by existing knowledge about the disease, including the number of known disease genes and their relationships within the existing data sources. We have used the concept of disease families in order to increase the number of known disease genes in each family. Some recent studies have considered relationships/similarities between diseases/phenotypes [32] and have utilized phenotype similarities in their gene prioritization approach [33]–[35]. We will investigate approaches to incorporate phenotype similarities into our framework.

## Supporting Information

Dataset S1 The informativeness measures for all disease families.
(XLSX)

Dataset S2 Top genes ranked by the three approaches on the PD dataset.
(XLSX)
